# Improving *Pigai* as an Automated Writing Evaluation System: Considerations for Refinement

**DOI:** 10.3389/fpsyg.2022.795725

**Published:** 2022-02-23

**Authors:** Xiaodong Zhang

**Affiliations:** School of English and International Studies, Beijing Foreign Studies University, Beijing, China

**Keywords:** automated writing evaluation system, English writing, university students, system refinement, context

## Introduction

**Figure 1:**
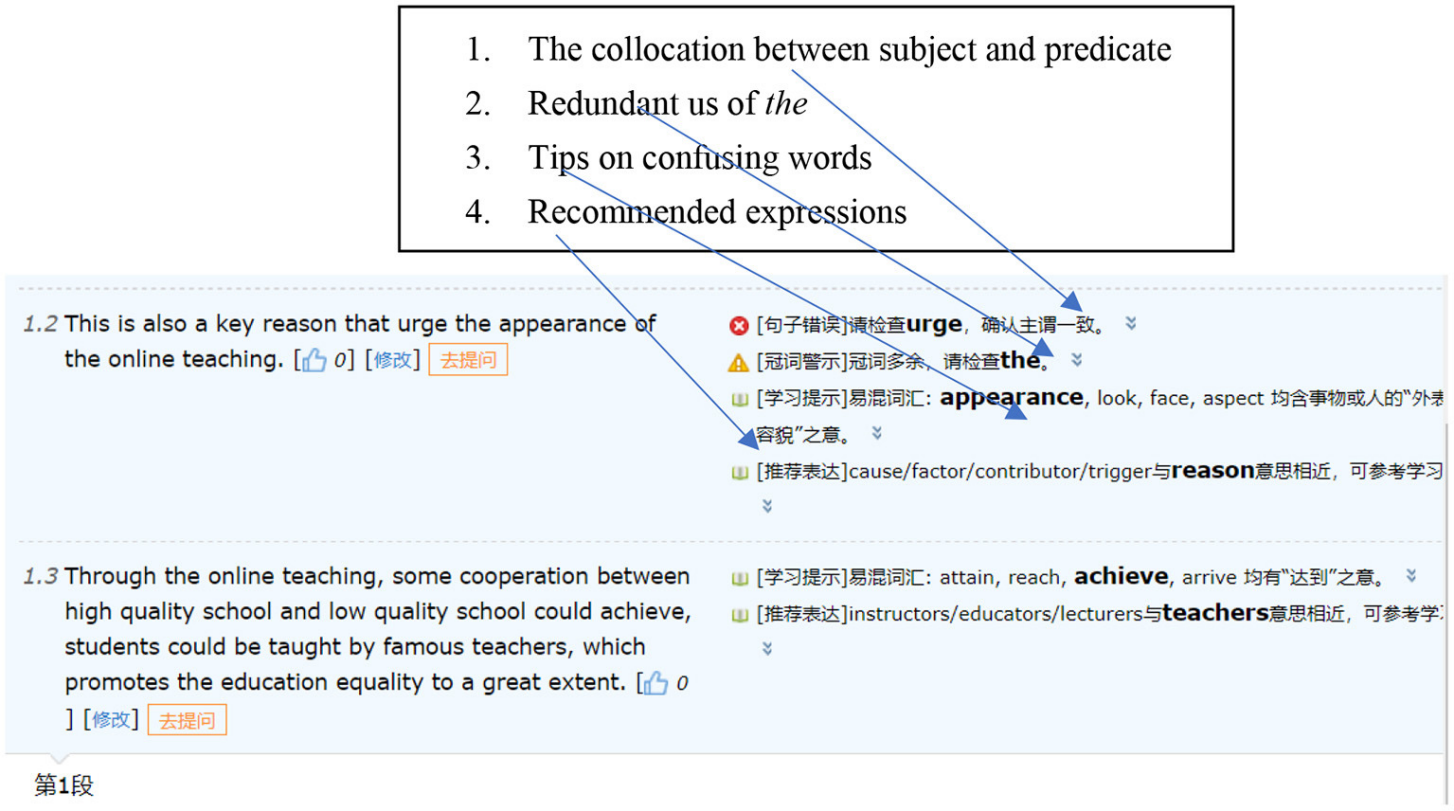
Sample feedback provided by *Pigai*.

The affordances offered by automated writing evaluation systems (AWES) have been widely reported (Wilson and Roscoe, 2020). In the university English-as-a-foreign-language (EFL) setting, *Pigai* has been widely used as one of AWES (Bai and Hu, 2016). With backup from multiple corpora (e.g., scored essays), *Pigai* could help users (e.g., student writers) quickly identify and correct grammatical errors and lexical inappropriateness at the sentence level, improving the accuracy of the evaluated essay (Bai and Hu, 2016). *Pigai* includes multiple dimensions of checking, such as typos, collocation, punctuation, article use, and word choice, whose assistance primarily lies at the sentence level (Yao, 2021; see also Figure 1).

However, effective and accurate writing is not merely static or mechanical (Derewianka and Jones, 2012). Instead, it is a meaning-making process, where the choice of language resources (including grammar and vocabulary) interacts with the context in which it is embedded—for example, the target audience—and with various cultural expectations (Schleppegrell, 2004). This means that *Pigai* needs to be additionally able to help diagnose such issues. For example, when a verb such as *knows* occurs with a third-person singular *she*, the verb may not be underlined by *Pigai*. Although the answer provided by AWES is correct for the sentence in terms of grammatical agreement between subject and verb in English, the automated feedback may be not correct when the writing is documenting an uncertain event. In other words, the appropriate sentence should be *she may know*. Existing negligence of such issues and inability to provide feedback regarding them are understandable. One primary reason may be a lack of technicians who understand the mechanism of effective writing in relation to writing process; if this is the case, technicians may not know how to incorporate context-based feedback into AWES. As Reinhardt and Oskoz (2021) also argued, in terms of technology-based teaching practices, we need to focus on situating “their uses and designs in commensurate theories of learning and pedagogy” (p. 2). To make better use of *Pigai* as one of AWES, the present paper discusses solutions and references for *Pigai* designers. It is hoped that *Pigai* designers may then be able to engage in empirical tests, collaborating with writing experts and taking actions to make further improvements of *Pigai*.

## Writing as a Meaning-Making Process and its Inclusion in Awes

It has been found that writing is a meaning-making process in response to context (Hinkel, 2002; Martin and Rose, 2006; Rosa and Hodgson-Drysdale, 2021). Therefore, in addition to ensuring sentential accuracy when combining language resources, optimal AWES, including *Pigai*, should consider whether the interrelationship between language choices and content is appropriate to a given context (Cotos, 2014).

In particular, the context includes (1) what a writing topic is about; (2) the social relationship a writer intends to construct with readers, as well as (3) how writing is to be presented (Schleppegrell, 2004). These contextual elements jointly exert their influence on the choice of language used (Hinkel, 2002; Martin and Rose, 2006). For example, in responding to a writing topic, topic-related vocabulary at the paragraph level or beyond has to be used, without which writing fails to convey a holistic idea. Logical connectors and logical appropriateness must be embodied among sentences. In response to social relationships, the tone of writing has to be appropriately represented through language choices, such as whether to be assertive or mitigated, or whether to be subjective or objective. For example, to avoid being assertive, modal verbs, quantifiers, or different types of reporting verbs need to be used. To present an objective tone, explicit use of evaluative language needs to be avoided. Citations or sources of information need to be presented. In response to how writing is to be presented, written sentences must be fluently conjugated. The language choices responsible for this include the use of synonyms, antonyms, hypernyms, pronoun references, or the simple repetition of the same word. Depending on the type of texts, the choice of language resources is further conditioned when realizing the social purpose of writing (Schleppegrell, 2004; Martin and Rose, 2006; Derewianka and Jones, 2012). For example, in science texts (informational texts), vocabulary may be found to feature technical terms and nominalization. The tone is objective, through the use of inanimate nouns, instead of pronouns (Hinkel, 2002; Schleppegrell, 2004; Martin and Rose, 2006; Rosa and Hodgson-Drysdale, 2021).

Taken together, this means that *Pigai* as an automated evaluation system needs to attend to this dynamic relationship along with ensuring the accuracy of sentences, thus comprehensively benefiting its users. In the following sections, potential strategies to achieve this are discussed.

## What Should Upgraded *PIGAI* Look Like?

An upgraded *Pigai* might include the dimension of plain language being used in the guiding instructions provided to users. Indeed, the *Pigai* should be to familiarize students with feedback giving and receiving, including how to understand and respond to the automated feedback provided by the system. Moreover, the addition to buttons that allow students to select the category of writing under which to upload their manuscripts would be needed. This would be a preliminary step preparing students for receiving feedback in relation to the context of their writing. Indeed, various types of writing differ in terms of expectations (Martin and Rose, 2006). Students' job, then, is to understand the types of writing they are composing and to upload them accordingly by clicking the correct button.

**Figure 2:**
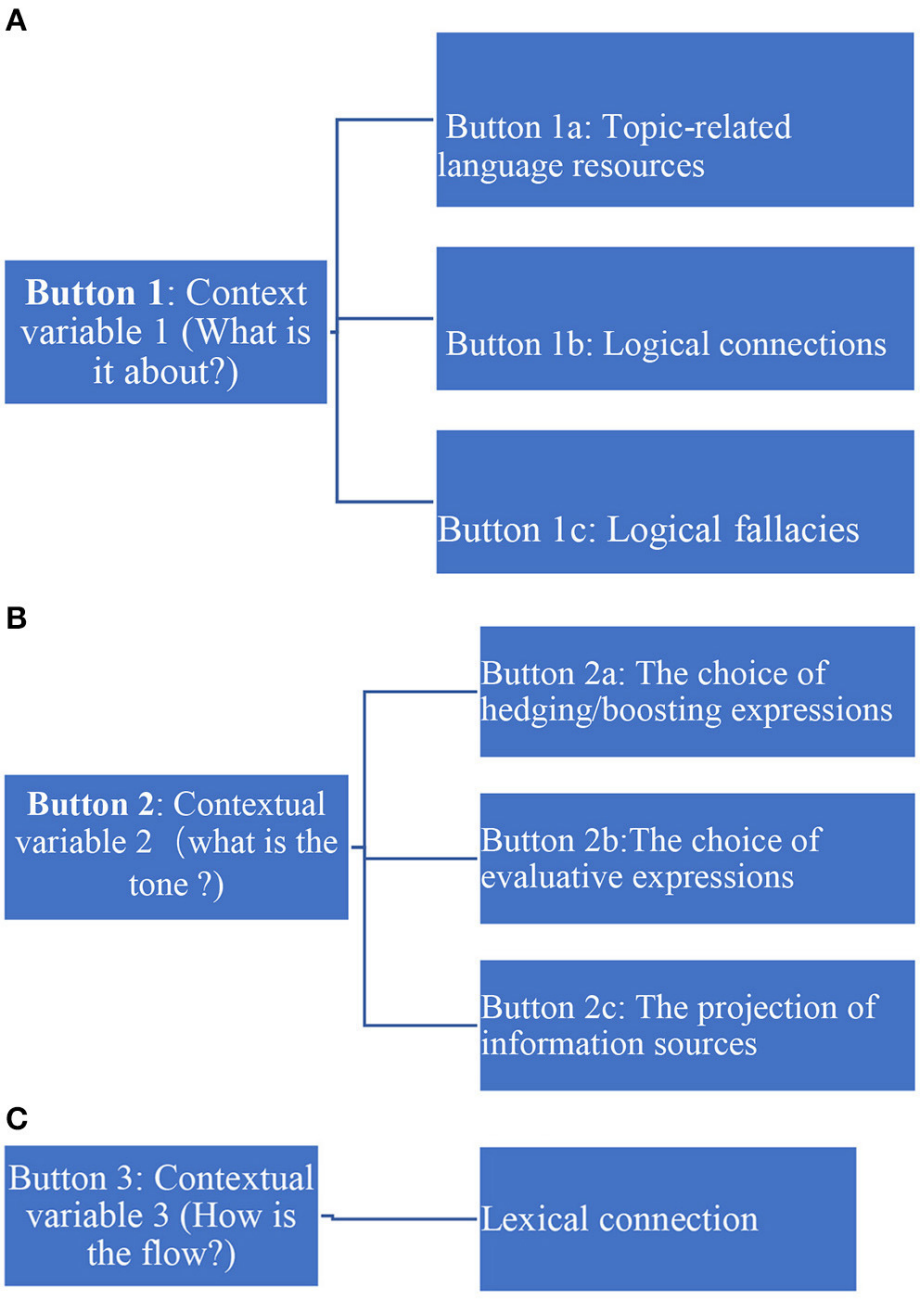
**(A)** Button 1 in relation to contextual variable 1. **(B)** Button 2 in relation to contextual variable 2. **(C)** Button 3 in relation to contextual variable 3.

The most important aspect of upgraded *Pigai* is able to provide automated feedback related to context. In particular, automated feedback could be designed in the following ways, through the addition of type-specific buttons (see Figures 2A–C, below).

Button 1 manages the relationship between contextual elements (what is it about?) and language resources. It will have three main sub-buttons. Button 1a, which diagnoses topic- related language resources. For example, nouns or verbs in the thesis and each topic sentence will be anchored as the basis. The nouns and verbs in the rest of the paragraph will be automatically analyzed at the semantical level. If there are sentences that do not have nouns and verbs related to the thesis and topic, feedback that could be provided is as follows: “Do the sentences still relate to the topic?”

Button 1b is responsible for logical relationships. Clicking Button 1b will help identify logical connectors. For sentences that do not have the connectors, feedback will include: “Please double-check the use of logical connectors. Button 1c should be programed with typical logical fallacies, which could be used to evaluate essays uploaded. Clicking the button will underline problematic areas with feedback: “Is the logical reasoning appropriate?”

When clicking Button 2, the tone of the writing in terms of generic demands will be checked. This is mainly comprised of three sub-buttons. With Button 2a, hedging words, such as modal verbs, and reporting verbs can be automatically identified and underlined. The accompanying feedback could be: “Is the information accurately delivered in line with literature? With Button 2b, evaluative language resources will be automatically identified, including adjectives or adverbs. The accompanying feedback is: “Does this type of writing favor (or not favor) the explicit use of evaluative resources?” Button 2c would be evaluating information source. Subject and reporting verbs, if any, will be concurrently identified by the system. Meanwhile, automated feedback will be provided on the sentences that do not have the structure: “Have parenthetical or in-text citations been provided?”

When clicking Button 3, the semantic relationship at the lexical level (such as hypernyms, antonyms, and references) can be automatically analyzed and marked. For Button 3, along with marks, automated feedback can be provided on the sentences that do not have such semantic relationship at the lexical level: “Are sentences lexically related?” With the underlined words and feedback generated by AWES, users may be reminded to attend to information fluency and make corrections, if possible.

The figures provide suggestions regarding upgrading *Pigai* as an automated writing system. However, it should be cautioned that the buttons are not intended to be exhaustive; they are intended for system developers' reference. Additional buttons may be needed in the actual design.

## Final Notes

The actual integration of contexts within *Pigai* may be challenging. The present paper suggests that writing instructors and software programmers make joint efforts to achieve such improvements as outlined here (Cotos, 2014). Writing instructors with the knowledge mentioned in the study could be invited to collaborate with software programmers, analyzing features of specific text types, upgrading existing systems, and helping students better diagnose their writing based on writing samples from similar contexts (Cotos, 2014). Financial support may be needed to connect these two groups of professionals in this task. This study suggests that the two parties could undertake promotions of the issues at academic conferences or with technology companies; if this were done, administrators and professionals in the fields of education and technology, respectively, might realize the vast educational value and economic potential of upgrading *Pigai* or other similar AWES, further facilitating the fruition of the upgraded system. Finally, AWES are not a panacea given the limitations of systems' capabilities. The proposed ideas may not be fully realized. Teachers will still need to offer individualized guidance to students when necessary (Bai and Hu, 2016). This also means the importance of offering teacher education programs for teachers whose classroom involves feedback. Indeed, teachers' professional training (e.g., their beliefs about how written feedback should be made, as well as their professional knowledge) plays a crucial role in either hampering or encouraging students' engagement with feedback (Lee and Mohebbi, 2021).

## Author Contributions

The author confirms being the sole contributor of this work and has approved it for publication.

## Conflict of Interest

The author declares that the research was conducted in the absence of any commercial or financial relationships that could be construed as a potential conflict of interest.

## Publisher's Note

All claims expressed in this article are solely those of the authors and do not necessarily represent those of their affiliated organizations, or those of the publisher, the editors and the reviewers. Any product that may be evaluated in this article, or claim that may be made by its manufacturer, is not guaranteed or endorsed by the publisher.
